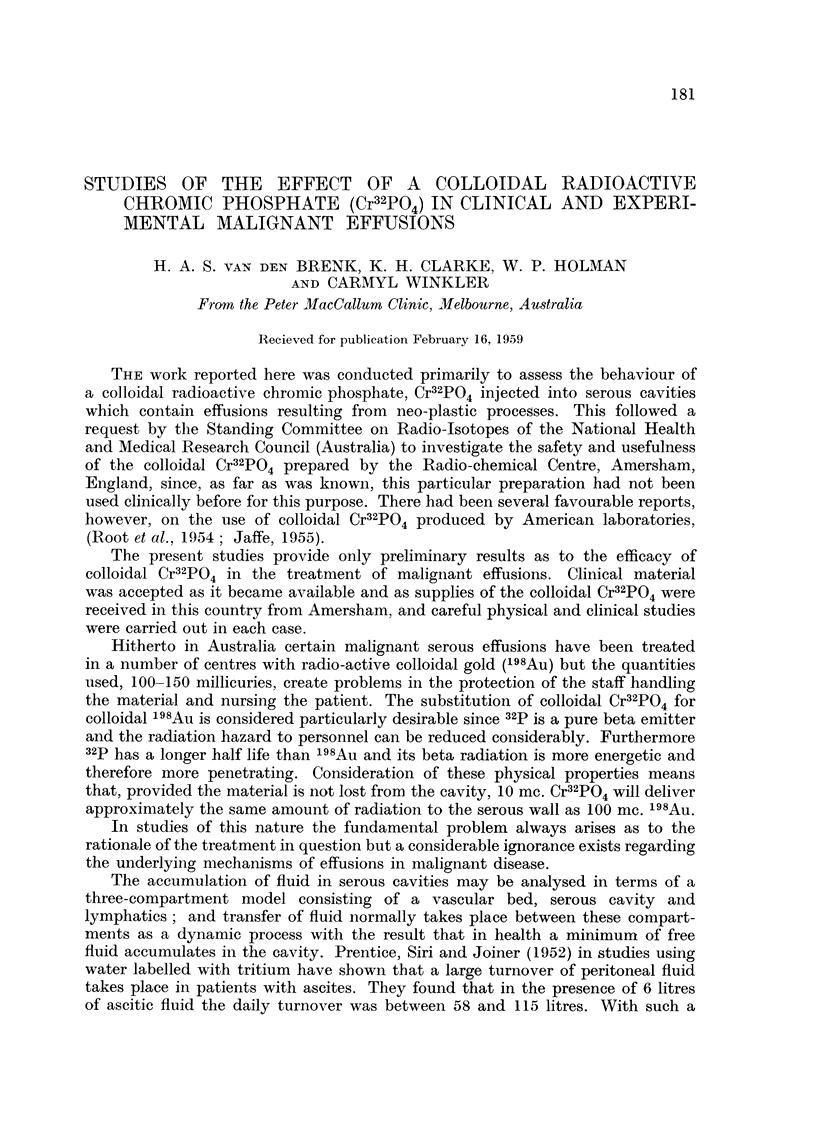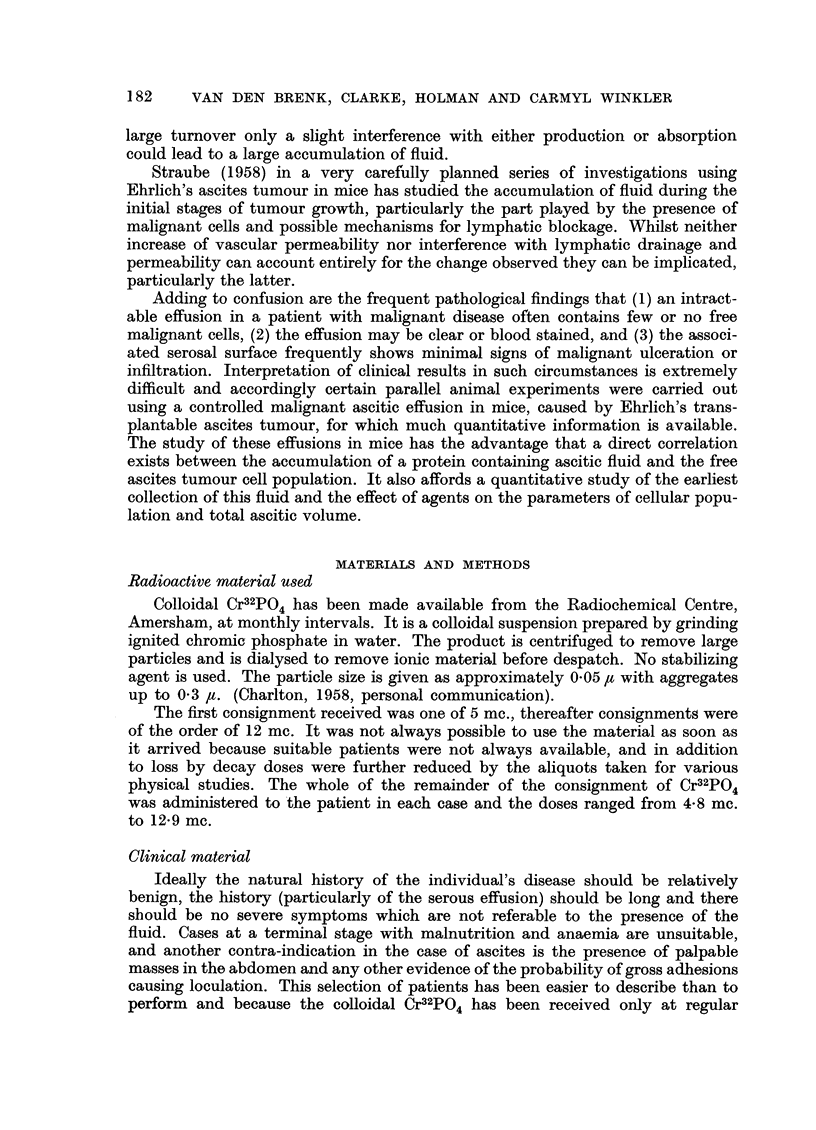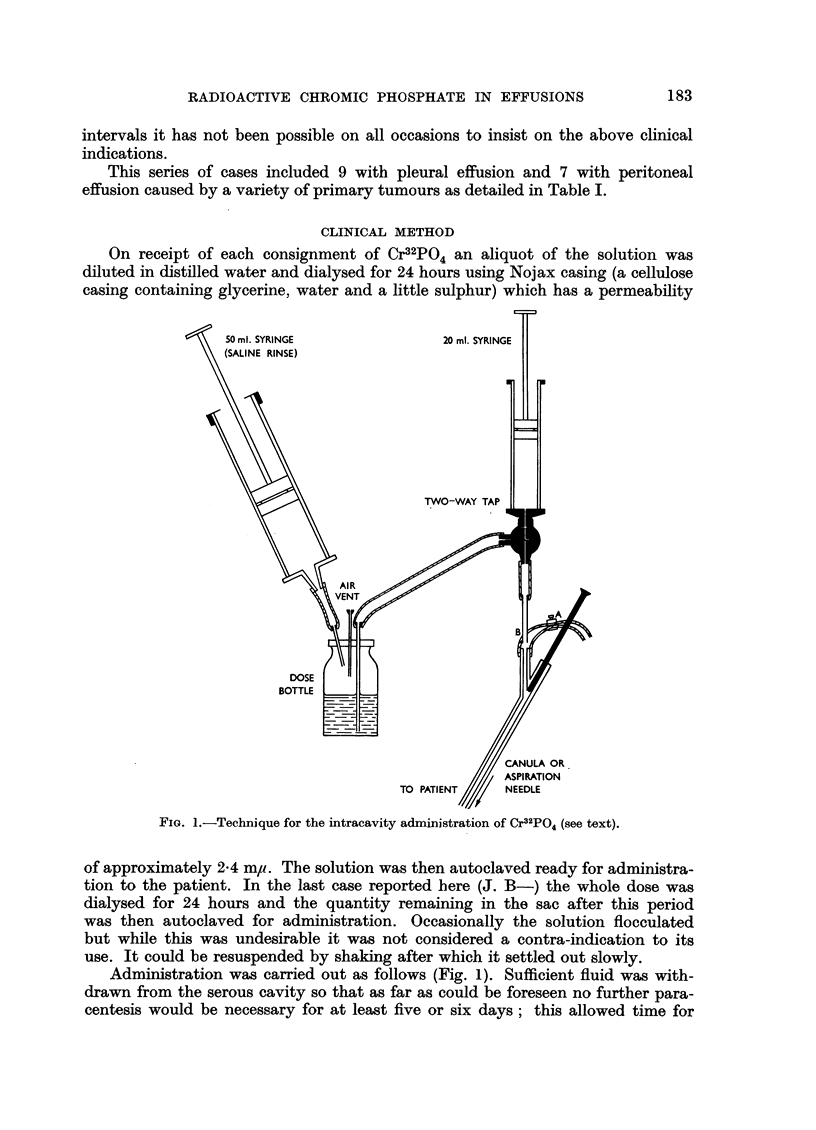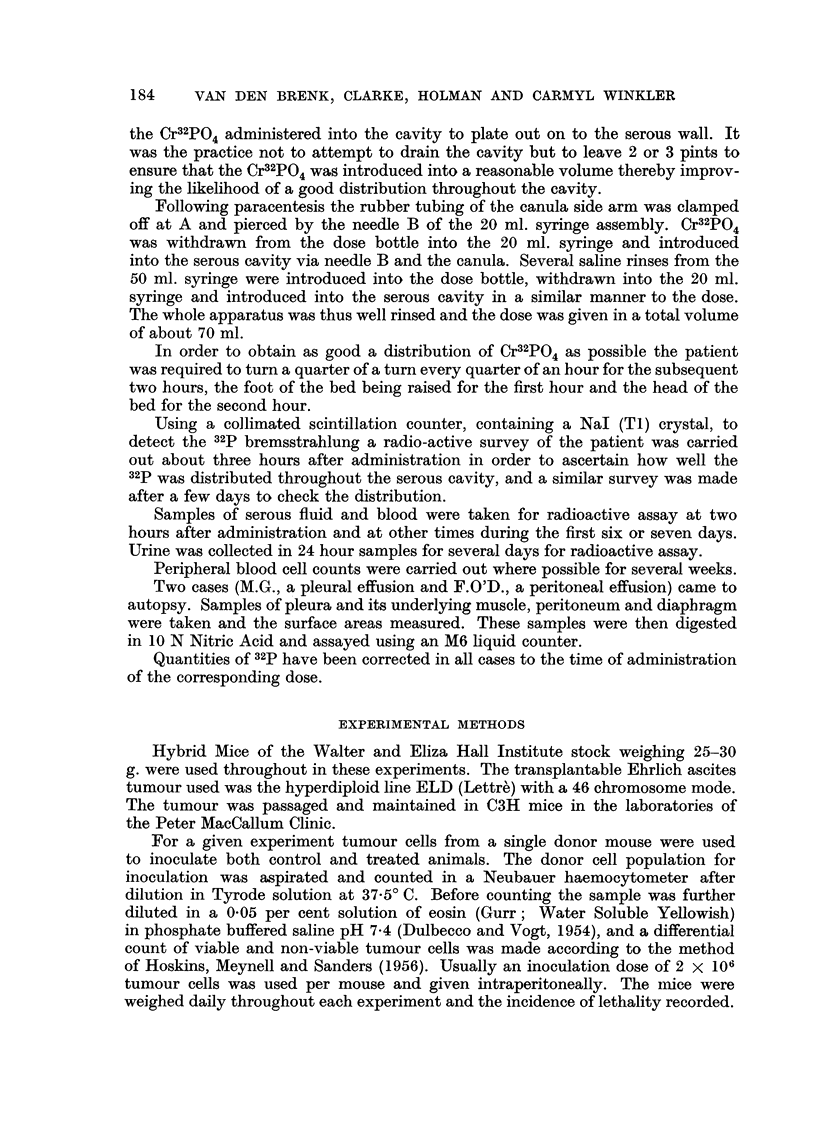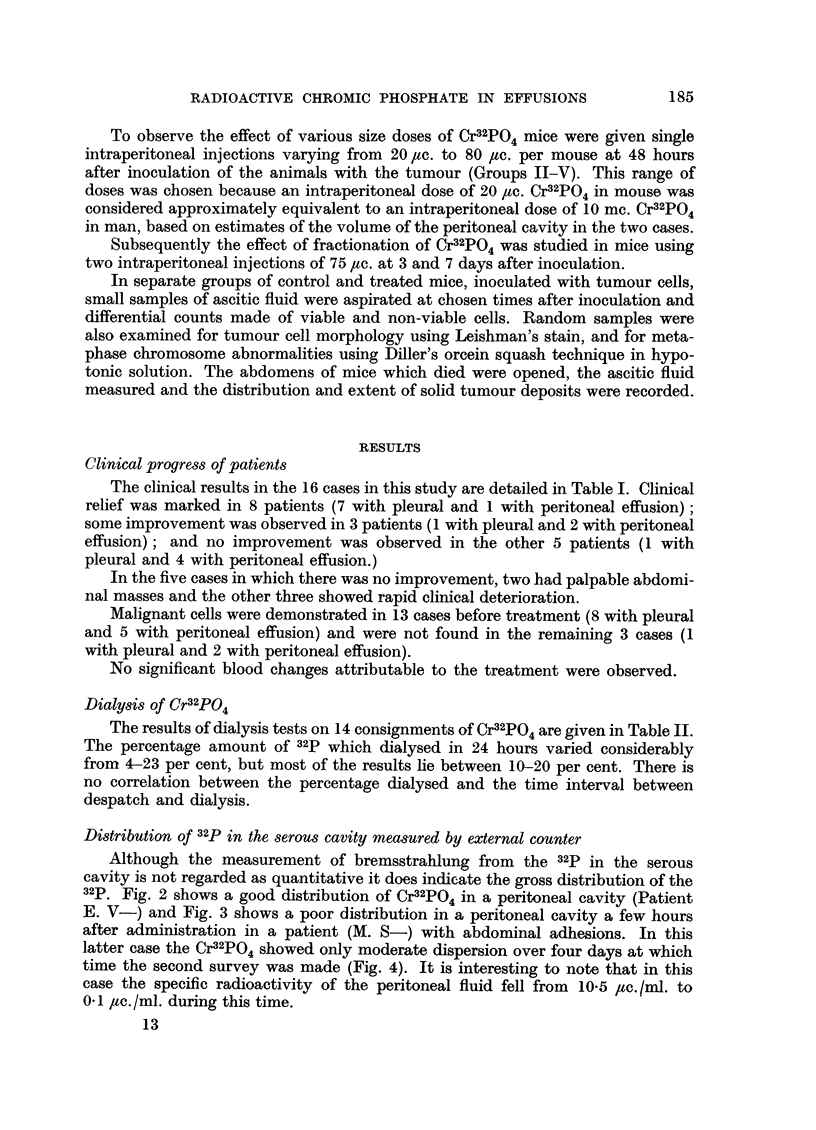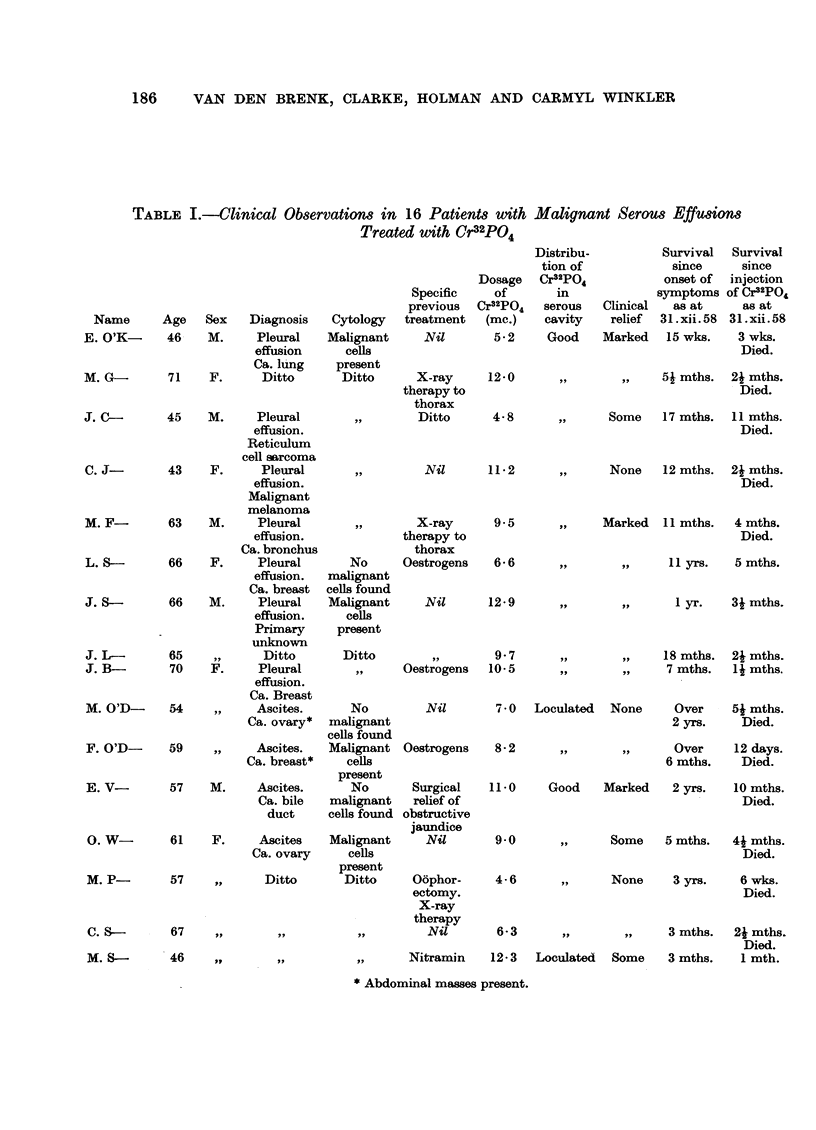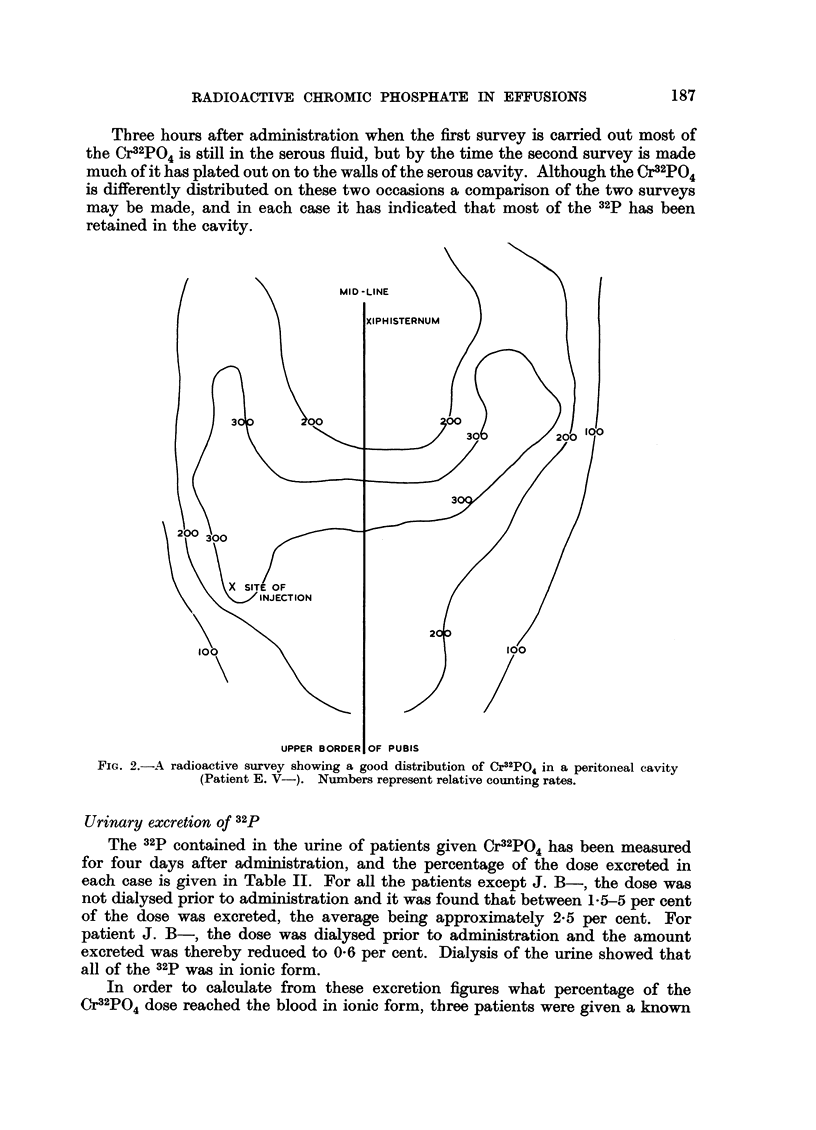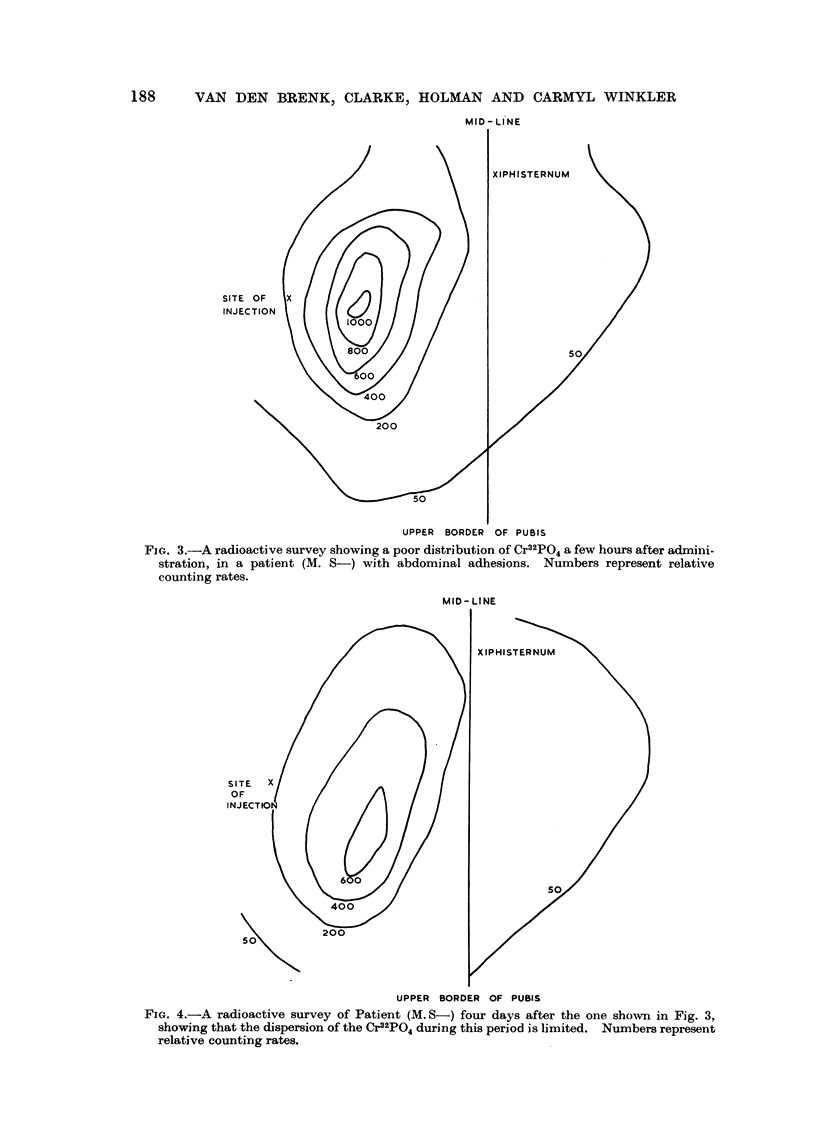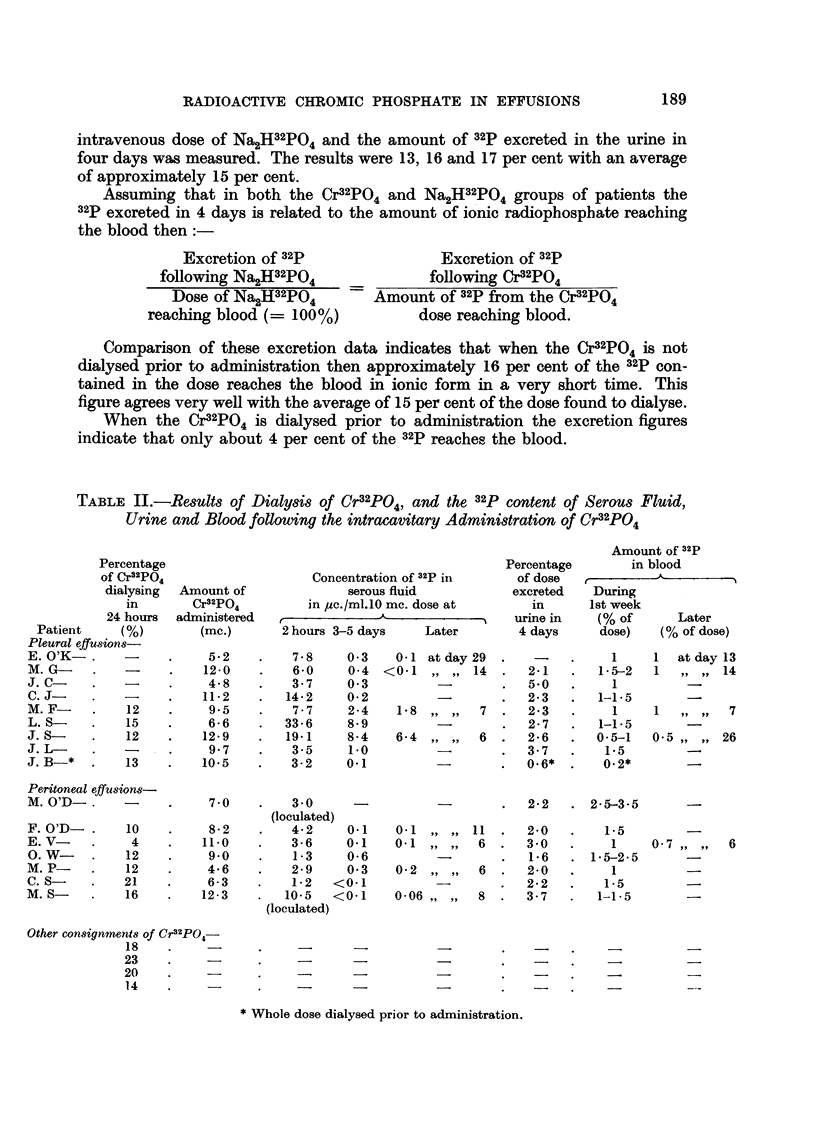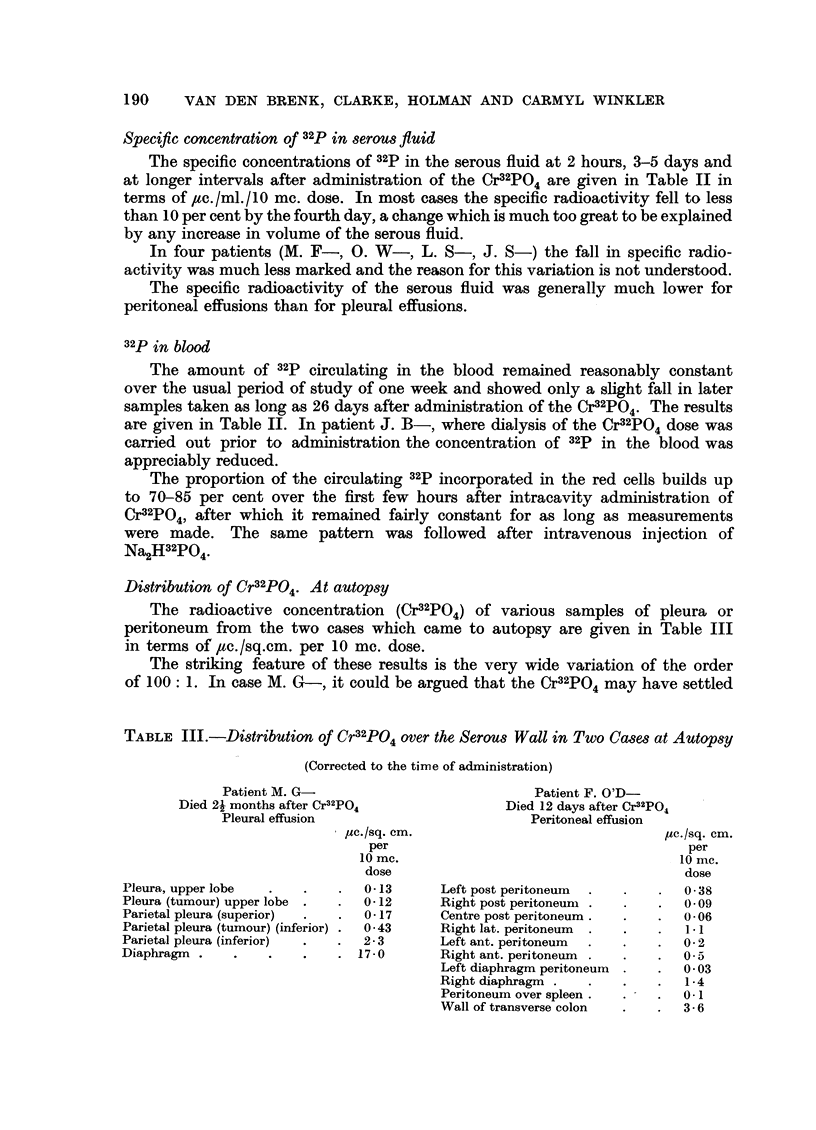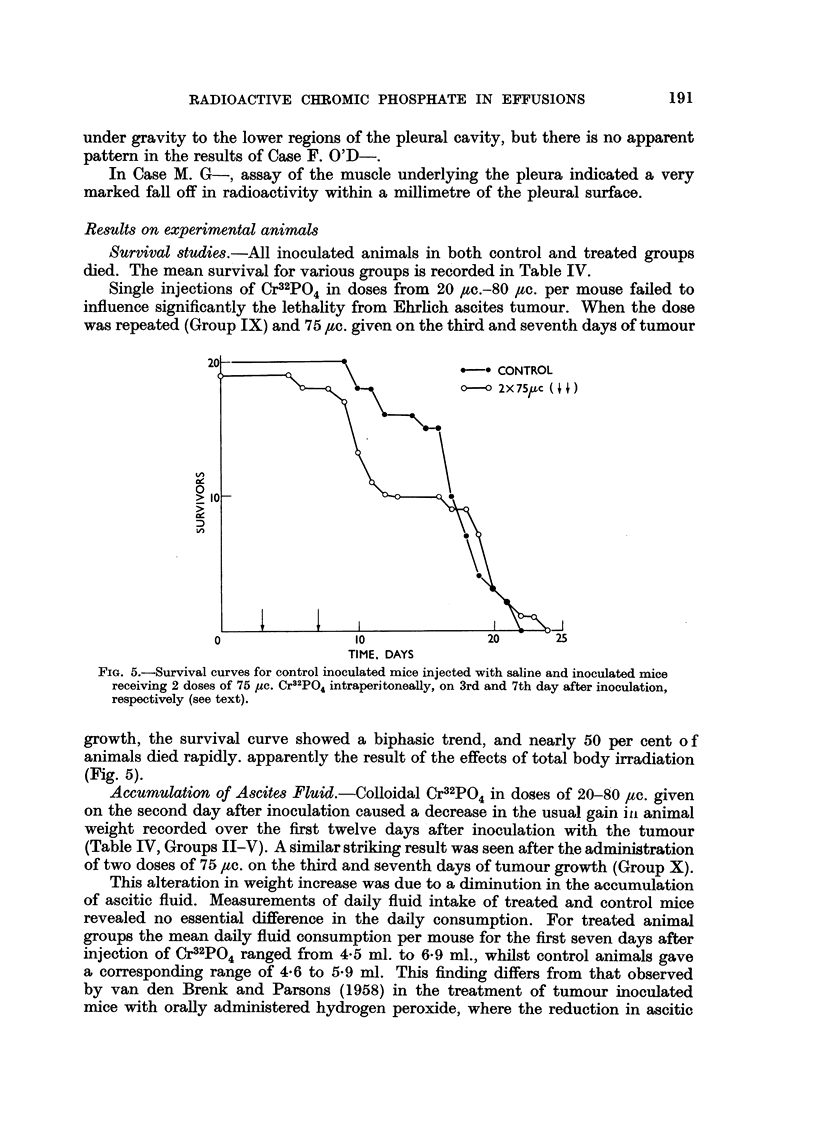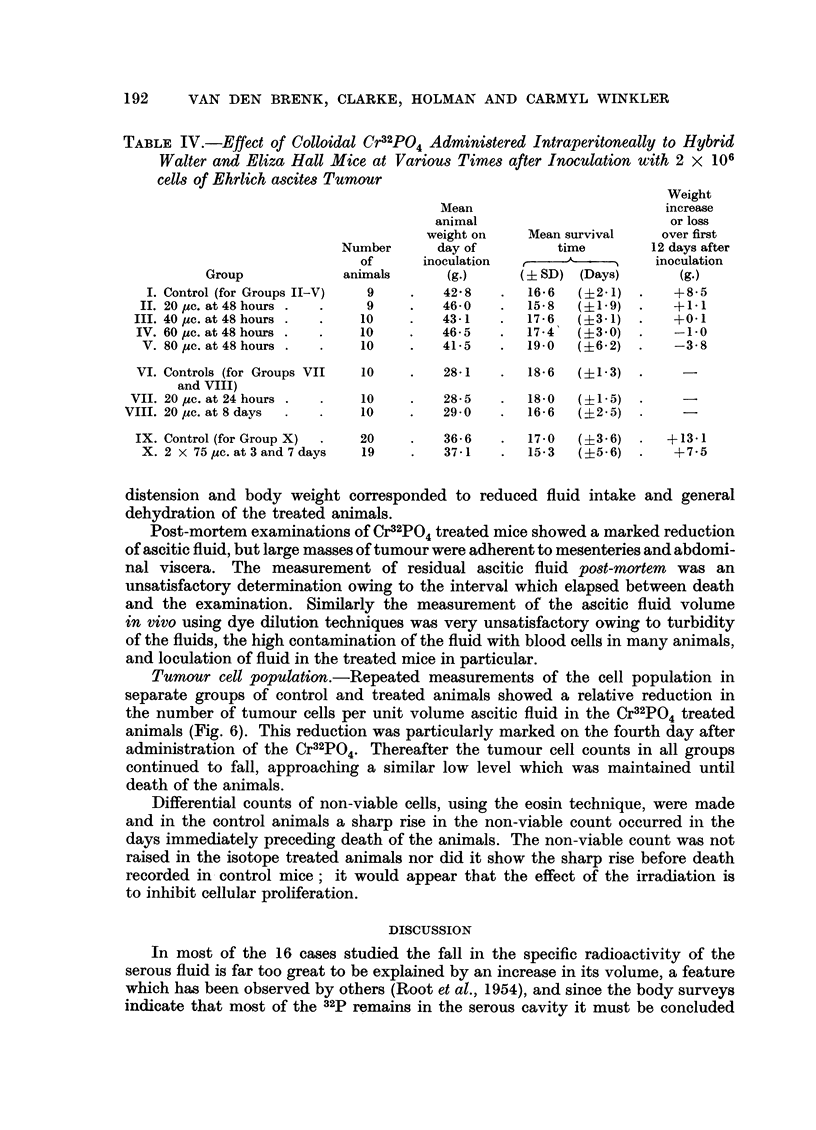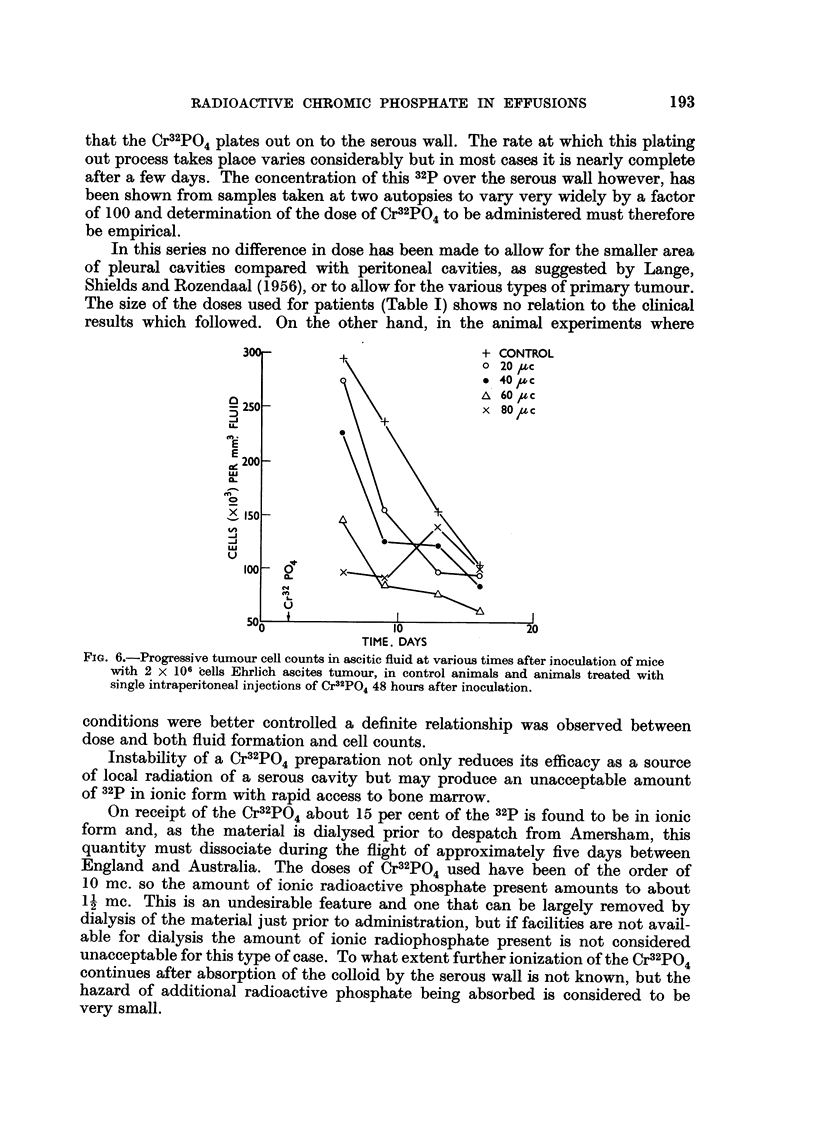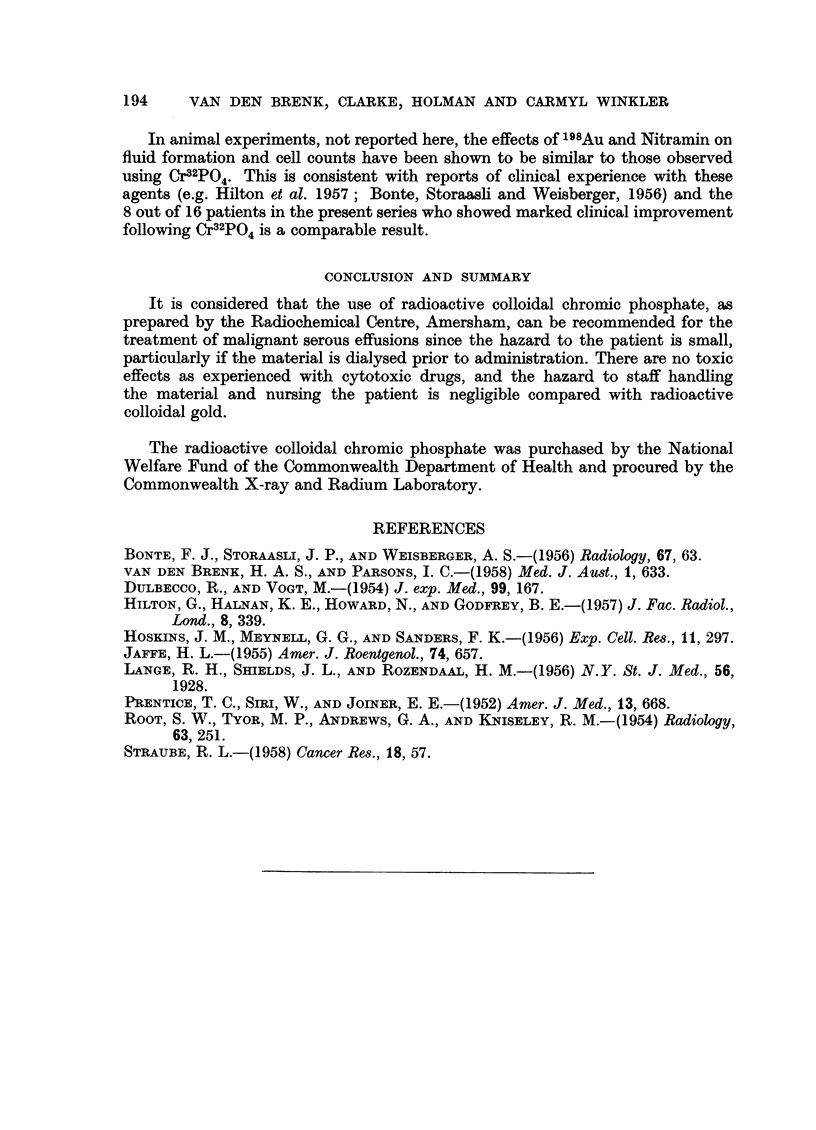# Studies of the Effect of a Colloidal Radioactive Chromic Phosphate (Cr^32^PO_4_) in Clinical and Experimental Malignant Effusions

**DOI:** 10.1038/bjc.1959.25

**Published:** 1959-06

**Authors:** H. A. S. van den Brenk, K. H. Clarke, W. P. Holman, Carmyl Winkler


### STUDIES OF THE EFFECT OF A COLLOIDAL RADIOACTIVE CHROMIC PHOSPHATE ($Cr^{32}PO_4$) IN CLINICAL AND EXPERIMENTAL MALIGNANT EFFUSIONS

H. A. S. VAN DEN BRENK, K. H. CLARKE, W. P. HOLMAN
AND CARMYL WINKLER

*From the Peter MacCallum Clinic, Melbourne, Australia*

Recieved for publication February 16, 1959

THE work reported here was conducted primarily to assess the behaviour of a colloidal radioactive chromic phosphate, $Cr^{32}PO_4$ injected into serous cavities which contain effusions resulting from neo-plastic processes. This followed a request by the Standing Committee on Radio-Isotopes of the National Health and Medical Research Council (Australia) to investigate the safety and usefulness of the colloidal $Cr^{32}PO_4$ prepared by the Radio-chemical Centre, Amersham, England, since, as far as was known, this particular preparation had not been used clinically before for this purpose. There had been several favourable reports, however, on the use of colloidal $Cr^{32}PO_4$ produced by American laboratories, (Root *et al.*, 1954 ; Jaffe, 1955).

The present studies provide only preliminary results as to the efficacy of colloidal $Cr^{32}PO_4$ in the treatment of malignant effusions. Clinical material was accepted as it became available and as supplies of the colloidal $Cr^{32}PO_4$ were received in this country from Amersham, and careful physical and clinical studies were carried out in each case.

Hitherto in Australia certain malignant serous effusions have been treated in a number of centres with radio-active colloidal gold ($^{198}Au$) but the quantities used, 100–150 millicuries, create problems in the protection of the staff handling the material and nursing the patient. The substitution of colloidal $Cr^{32}PO_4$ for colloidal $^{198}Au$ is considered particularly desirable since $^{32}P$ is a pure beta emitter and the radiation hazard to personnel can be reduced considerably. Furthermore $^{32}P$ has a longer half life than $^{198}Au$ and its beta radiation is more energetic and therefore more penetrating. Consideration of these physical properties means that, provided the material is not lost from the cavity, 10 mc. $Cr^{32}PO_4$ will deliver approximately the same amount of radiation to the serous wall as 100 mc. $^{198}Au$.

In studies of this nature the fundamental problem always arises as to the rationale of the treatment in question but a considerable ignorance exists regarding the underlying mechanisms of effusions in malignant disease.

The accumulation of fluid in serous cavities may be analysed in terms of a three-compartment model consisting of a vascular bed, serous cavity and lymphatics ; and transfer of fluid normally takes place between these compartments as a dynamic process with the result that in health a minimum of free fluid accumulates in the cavity. Prentice, Siri and Joiner (1952) in studies using water labelled with tritium have shown that a large turnover of peritoneal fluid takes place in patients with ascites. They found that in the presence of 6 litres of ascitic fluid the daily turnover was between 58 and 115 litres. With such a large turnover only a slight interference with either production or absorption could lead to a large accumulation of fluid.

Straube (1958) in a very carefully planned series of investigations using Ehrlich's ascites tumour in mice has studied the accumulation of fluid during the initial stages of tumour growth, particularly the part played by the presence of malignant cells and possible mechanisms for lymphatic blockage. Whilst neither increase of vascular permeability nor interference with lymphatic drainage and permeability can account entirely for the change observed they can be implicated, particularly the latter.

Adding to confusion are the frequent pathological findings that (1) an intractable effusion in a patient with malignant disease often contains few or no free malignant cells, (2) the effusion may be clear or blood stained, and (3) the associated serosal surface frequently shows minimal signs of malignant ulceration or infiltration. Interpretation of clinical results in such circumstances is extremely difficult and accordingly certain parallel animal experiments were carried out using a controlled malignant ascitic effusion in mice, caused by Ehrlich's transplantable ascites tumour, for which much quantitative information is available. The study of these effusions in mice has the advantage that a direct correlation exists between the accumulation of a protein containing ascitic fluid and the free ascites tumour cell population. It also affords a quantitative study of the earliest collection of this fluid and the effect of agents on the parameters of cellular population and total ascitic volume.

### MATERIALS AND METHODS

### *Radioactive material used*

Colloidal $Cr^{32}PO_4$ has been made available from the Radiochemical Centre, Amersham, at monthly intervals. It is a colloidal suspension prepared by grinding ignited chromic phosphate in water. The product is centrifuged to remove large particles and is dialysed to remove ionic material before despatch. No stabilizing agent is used. The particle size is given as approximately 0·05 $\mu$ with aggregates up to 0·3 $\mu$. (Charlton, 1958, personal communication).

The first consignment received was one of 5 mc., thereafter consignments were of the order of 12 mc. It was not always possible to use the material as soon as it arrived because suitable patients were not always available, and in addition to loss by decay doses were further reduced by the aliquots taken for various physical studies. The whole of the remainder of the consignment of $Cr^{32}PO_4$ was administered to the patient in each case and the doses ranged from 4·8 mc. to 12·9 mc.

### *Clinical material*

Ideally the natural history of the individual's disease should be relatively benign, the history (particularly of the serous effusion) should be long and there should be no severe symptoms which are not referable to the presence of the fluid. Cases at a terminal stage with malnutrition and anaemia are unsuitable, and another contra-indication in the case of ascites is the presence of palpable masses in the abdomen and any other evidence of the probability of gross adhesions causing loculation. This selection of patients has been easier to describe than to perform and because the colloidal $Cr^{32}PO_4$ has been received only at regular intervals it has not been possible on all occasions to insist on the above clinical indications.

This series of cases included 9 with pleural effusion and 7 with peritoneal effusion caused by a variety of primary tumours as detailed in Table I.

### CLINICAL METHOD

On receipt of each consignment of $Cr^{32}PO_4$ an aliquot of the solution was diluted in distilled water and dialysed for 24 hours using Nojax casing (a cellulose casing containing glycerine, water and a little sulphur) which has a permeability

FIG. 1.—Technique for the intracavity administration of $Cr^{32}PO_4$ (see text).

of approximately 2·4 m$\mu$. The solution was then autoclaved ready for administration to the patient. In the last case reported here (J. B—) the whole dose was dialysed for 24 hours and the quantity remaining in the sac after this period was then autoclaved for administration. Occasionally the solution flocculated but while this was undesirable it was not considered a contra-indication to its use. It could be resuspended by shaking after which it settled out slowly.

Administration was carried out as follows (Fig. 1). Sufficient fluid was withdrawn from the serous cavity so that as far as could be foreseen no further paracentesis would be necessary for at least five or six days ; this allowed time for the $Cr^{32}PO_4$ administered into the cavity to plate out on to the serous wall. It was the practice not to attempt to drain the cavity but to leave 2 or 3 pints to ensure that the $Cr^{32}PO_4$ was introduced into a reasonable volume thereby improving the likelihood of a good distribution throughout the cavity.

Following paracentesis the rubber tubing of the canula side arm was clamped off at A and pierced by the needle B of the 20 ml. syringe assembly. $Cr^{32}PO_4$ was withdrawn from the dose bottle into the 20 ml. syringe and introduced into the serous cavity via needle B and the canula. Several saline rinses from the 50 ml. syringe were introduced into the dose bottle, withdrawn into the 20 ml. syringe and introduced into the serous cavity in a similar manner to the dose. The whole apparatus was thus well rinsed and the dose was given in a total volume of about 70 ml.

In order to obtain as good a distribution of $Cr^{32}PO_4$ as possible the patient was required to turn a quarter of a turn every quarter of an hour for the subsequent two hours, the foot of the bed being raised for the first hour and the head of the bed for the second hour.

Using a collimated scintillation counter, containing a NaI (Tl) crystal, to detect the $^{32}P$ bremsstrahlung a radio-active survey of the patient was carried out about three hours after administration in order to ascertain how well the $^{32}P$ was distributed throughout the serous cavity, and a similar survey was made after a few days to check the distribution.

Samples of serous fluid and blood were taken for radioactive assay at two hours after administration and at other times during the first six or seven days. Urine was collected in 24 hour samples for several days for radioactive assay.

Peripheral blood cell counts were carried out where possible for several weeks.

Two cases (M.G., a pleural effusion and F.O'D., a peritoneal effusion) came to autopsy. Samples of pleura and its underlying muscle, peritoneum and diaphragm were taken and the surface areas measured. These samples were then digested in 10 N Nitric Acid and assayed using an M6 liquid counter.

Quantities of $^{32}P$ have been corrected in all cases to the time of administration of the corresponding dose.

### EXPERIMENTAL METHODS

Hybrid Mice of the Walter and Eliza Hall Institute stock weighing 25–30 g. were used throughout in these experiments. The transplantable Ehrlich ascites tumour used was the hyperdiploid line ELD (Lettrè) with a 46 chromosome mode. The tumour was passaged and maintained in C3H mice in the laboratories of the Peter MacCallum Clinic.

For a given experiment tumour cells from a single donor mouse were used to inoculate both control and treated animals. The donor cell population for inoculation was aspirated and counted in a Neubauer haemocytometer after dilution in Tyrode solution at 37·5° C. Before counting the sample was further diluted in a 0·05 per cent solution of eosin (Gurr; Water Soluble Yellowish) in phosphate buffered saline pH 7·4 (Dulbecco and Vogt, 1954), and a differential count of viable and non-viable tumour cells was made according to the method of Hoskins, Meynell and Sanders (1956). Usually an inoculation dose of $2 \times 10^6$ tumour cells was used per mouse and given intraperitoneally. The mice were weighed daily throughout each experiment and the incidence of lethality recorded.

To observe the effect of various size doses of $Cr^{32}PO_4$ mice were given single intraperitoneal injections varying from 20 $\mu$c. to 80 $\mu$c. per mouse at 48 hours after inoculation of the animals with the tumour (Groups II–V). This range of doses was chosen because an intraperitoneal dose of 20 $\mu$c. $Cr^{32}PO_4$ in mouse was considered approximately equivalent to an intraperitoneal dose of 10 mc. $Cr^{32}PO_4$ in man, based on estimates of the volume of the peritoneal cavity in the two cases.

Subsequently the effect of fractionation of $Cr^{32}PO_4$ was studied in mice using two intraperitoneal injections of 75 $\mu$c. at 3 and 7 days after inoculation.

In separate groups of control and treated mice, inoculated with tumour cells, small samples of ascitic fluid were aspirated at chosen times after inoculation and differential counts made of viable and non-viable cells. Random samples were also examined for tumour cell morphology using Leishman's stain, and for metaphase chromosome abnormalities using Diller's orcein squash technique in hypotonic solution. The abdomens of mice which died were opened, the ascitic fluid measured and the distribution and extent of solid tumour deposits were recorded.

### RESULTS

### *Clinical progress of patients*

The clinical results in the 16 cases in this study are detailed in Table I. Clinical relief was marked in 8 patients (7 with pleural and 1 with peritoneal effusion); some improvement was observed in 3 patients (1 with pleural and 2 with peritoneal effusion); and no improvement was observed in the other 5 patients (1 with pleural and 4 with peritoneal effusion.)

In the five cases in which there was no improvement, two had palpable abdominal masses and the other three showed rapid clinical deterioration.

Malignant cells were demonstrated in 13 cases before treatment (8 with pleural and 5 with peritoneal effusion) and were not found in the remaining 3 cases (1 with pleural and 2 with peritoneal effusion).

No significant blood changes attributable to the treatment were observed.

### *Dialysis of* $Cr^{32}PO_4$

The results of dialysis tests on 14 consignments of $Cr^{32}PO_4$ are given in Table II. The percentage amount of $^{32}P$ which dialysed in 24 hours varied considerably from 4–23 per cent, but most of the results lie between 10–20 per cent. There is no correlation between the percentage dialysed and the time interval between despatch and dialysis.

### *Distribution of* $^{32}P$ *in the serous cavity measured by external counter*

Although the measurement of bremsstrahlung from the $^{32}P$ in the serous cavity is not regarded as quantitative it does indicate the gross distribution of the $^{32}P$. Fig. 2 shows a good distribution of $Cr^{32}PO_4$ in a peritoneal cavity (Patient E. V—) and Fig. 3 shows a poor distribution in a peritoneal cavity a few hours after administration in a patient (M. S—) with abdominal adhesions. In this latter case the $Cr^{32}PO_4$ showed only moderate dispersion over four days at which time the second survey was made (Fig. 4). It is interesting to note that in this case the specific radioactivity of the peritoneal fluid fell from 10·5 $\mu$c./ml. to 0·1 $\mu$c./ml. during this time.

TABLE I.—*Clinical Observations in 16 Patients with Malignant Serous Effusions Treated with $Cr^{32}PO_4$*

| Name | Age | Sex | Diagnosis | Cytology | Specific previous treatment | Dosage of $Cr^{32}PO_4$ (mc.) | Distribution of $Cr^{32}PO_4$ in serous cavity | Clinical relief | Survival since onset of symptoms as at 31.xii.58 | Survival since injection of $Cr^{32}PO_4$ as at 31.xii.58 |
|---|---|---|---|---|---|---|---|---|---|---|
| E. O'K— | 46 | M. | Pleural effusion Ca. lung | Malignant cells present | *Nil* | 5·2 | Good | Marked | 15 wks. | 3 wks. Died. |
| M. G— | 71 | F. | Ditto | Ditto | X-ray therapy to thorax | 12·0 | ,, | ,, | 5½ mths. | 2½ mths. Died. |
| J. C— | 45 | M. | Pleural effusion. Reticulum cell sarcoma | ,, | Ditto | 4·8 | ,, | Some | 17 mths. | 11 mths. Died. |
| C. J— | 43 | F. | Pleural effusion. Malignant melanoma | ,, | *Nil* | 11·2 | ,, | None | 12 mths. | 2½ mths. Died. |
| M. F— | 63 | M. | Pleural effusion. Ca. bronchus | ,, | X-ray therapy to thorax | 9·5 | ,, | Marked | 11 mths. | 4 mths. Died. |
| L. S— | 66 | F. | Pleural effusion. Ca. breast | No malignant cells found | Oestrogens | 6·6 | ,, | ,, | 11 yrs. | 5 mths. |
| J. S— | 66 | M. | Pleural effusion. Primary unknown | Malignant cells present | *Nil* | 12·9 | ,, | ,, | 1 yr. | 3½ mths. |
| J. L— | 65 | ,, | Ditto | Ditto | ,, | 9·7 | ,, | ,, | 18 mths. | 2½ mths. |
| J. B— | 70 | F. | Pleural effusion. Ca. Breast | ,, | Oestrogens | 10·5 | ,, | ,, | 7 mths. | 1½ mths. |
| M. O'D— | 54 | ,, | Ascites. Ca. ovary* | No malignant cells found | *Nil* | 7·0 | Loculated | None | Over 2 yrs. | 5½ mths. Died. |
| F. O'D— | 59 | ,, | Ascites. Ca. breast* | Malignant cells present | Oestrogens | 8·2 | ,, | ,, | Over 6 mths. | 12 days. Died. |
| E. V— | 57 | M. | Ascites. Ca. bile duct | No malignant cells found | Surgical relief of obstructive jaundice | 11·0 | Good | Marked | 2 yrs. | 10 mths. Died. |
| O. W— | 61 | F. | Ascites Ca. ovary | Malignant cells present | *Nil* | 9·0 | ,, | Some | 5 mths. | 4½ mths. Died. |
| M. P— | 57 | ,, | Ditto | Ditto | Oöphorectomy. X-ray therapy | 4·6 | ,, | None | 3 yrs. | 6 wks. Died. |
| C. S— | 67 | ,, | ,, | ,, | *Nil* | 6·3 | ,, | ,, | 3 mths. | 2½ mths. Died. |
| M. S— | 46 | ,, | ,, | ,, | Nitramin | 12·3 | Loculated | Some | 3 mths. | 1 mth. |

* Abdominal masses present.

Three hours after administration when the first survey is carried out most of the $Cr^{32}PO_4$ is still in the serous fluid, but by the time the second survey is made much of it has plated out on to the walls of the serous cavity. Although the $Cr^{32}PO_4$ is differently distributed on these two occasions a comparison of the two surveys may be made, and in each case it has indicated that most of the $^{32}P$ has been retained in the cavity.

FIG. 2.—A radioactive survey showing a good distribution of $Cr^{32}PO_4$ in a peritoneal cavity (Patient E. V—). Numbers represent relative counting rates.

*Urinary excretion of $^{32}P$*

The $^{32}P$ contained in the urine of patients given $Cr^{32}PO_4$ has been measured for four days after administration, and the percentage of the dose excreted in each case is given in Table II. For all the patients except J. B—, the dose was not dialysed prior to administration and it was found that between 1·5–5 per cent of the dose was excreted, the average being approximately 2·5 per cent. For patient J. B—, the dose was dialysed prior to administration and the amount excreted was thereby reduced to 0·6 per cent. Dialysis of the urine showed that all of the $^{32}P$ was in ionic form.

In order to calculate from these excretion figures what percentage of the $Cr^{32}PO_4$ dose reached the blood in ionic form, three patients were given a known

FIG. 3.—A radioactive survey showing a poor distribution of $Cr^{32}PO_4$ a few hours after administration, in a patient (M. S—) with abdominal adhesions. Numbers represent relative counting rates.

FIG. 4.—A radioactive survey of Patient (M.S—) four days after the one shown in Fig. 3, showing that the dispersion of the $Cr^{32}PO_4$ during this period is limited. Numbers represent relative counting rates.

intravenous dose of $Na_2H^{32}PO_4$ and the amount of $^{32}P$ excreted in the urine in four days was measured. The results were 13, 16 and 17 per cent with an average of approximately 15 per cent.

Assuming that in both the $Cr^{32}PO_4$ and $Na_2H^{32}PO_4$ groups of patients the $^{32}P$ excreted in 4 days is related to the amount of ionic radiophosphate reaching the blood then :—

$$\frac{\text{Excretion of } ^{32}P \text{ following } Na_2H^{32}PO_4}{\text{Dose of } Na_2H^{32}PO_4 \text{ reaching blood } (= 100\%)} = \frac{\text{Excretion of } ^{32}P \text{ following } Cr^{32}PO_4}{\text{Amount of } ^{32}P \text{ from the } Cr^{32}PO_4 \text{ dose reaching blood.}}$$

Comparison of these excretion data indicates that when the $Cr^{32}PO_4$ is not dialysed prior to administration then approximately 16 per cent of the $^{32}P$ contained in the dose reaches the blood in ionic form in a very short time. This figure agrees very well with the average of 15 per cent of the dose found to dialyse.

When the $Cr^{32}PO_4$ is dialysed prior to administration the excretion figures indicate that only about 4 per cent of the $^{32}P$ reaches the blood.

TABLE II.—*Results of Dialysis of $Cr^{32}PO_4$, and the $^{32}P$ content of Serous Fluid, Urine and Blood following the intracavitary Administration of $Cr^{32}PO_4$*

| Patient | Percentage of $Cr^{32}PO_4$ dialysing in 24 hours (%) | Amount of $Cr^{32}PO_4$ administered (mc.) | Concentration of $^{32}P$ in serous fluid in μc./ml.10 mc. dose at: 2 hours | 3–5 days | Later | Percentage of dose excreted in urine in 4 days | Amount of $^{32}P$ in blood: During 1st week (% of dose) | Later (% of dose) |
|---|---|---|---|---|---|---|---|---|
| *Pleural effusions—* | | | | | | | | |
| E. O'K— | — | 5·2 | 7·8 | 0·3 | 0·1 at day 29 | — | 1 | 1 at day 13 |
| M. G— | — | 12·0 | 6·0 | 0·4 | <0·1 ,, ,, 14 | 2·1 | 1·5–2 | 1 ,, ,, 14 |
| J. C— | — | 4·8 | 3·7 | 0·3 | — | 5·0 | 1 | — |
| C. J— | — | 11·2 | 14·2 | 0·2 | — | 2·3 | 1–1·5 | — |
| M. F— | 12 | 9·5 | 7·7 | 2·4 | 1·8 ,, ,, 7 | 2·3 | 1 | 1 ,, ,, 7 |
| L. S— | 15 | 6·6 | 33·6 | 8·9 | — | 2·7 | 1–1·5 | — |
| J. S— | 12 | 12·9 | 19·1 | 8·4 | 6·4 ,, ,, 6 | 2·6 | 0·5–1 | 0·5 ,, ,, 26 |
| J. L— | — | 9·7 | 3·5 | 1·0 | — | 3·7 | 1·5 | — |
| J. B—* | 13 | 10·5 | 3·2 | 0·1 | — | 0·6* | 0·2* | — |
| *Peritoneal effusions—* | | | | | | | | |
| M. O'D— | — | 7·0 | 3·0 (loculated) | — | — | 2·2 | 2·5–3·5 | — |
| F. O'D— | 10 | 8·2 | 4·2 | 0·1 | 0·1 ,, ,, 11 | 2·0 | 1·5 | — |
| E. V— | 4 | 11·0 | 3·6 | 0·1 | 0·1 ,, ,, 6 | 3·0 | 1 | 0·7 ,, ,, 6 |
| O. W— | 12 | 9·0 | 1·3 | 0·6 | — | 1·6 | 1·5–2·5 | — |
| M. P— | 12 | 4·6 | 2·9 | 0·3 | 0·2 ,, ,, 6 | 2·0 | 1 | — |
| C. S— | 21 | 6·3 | 1·2 | <0·1 | — | 2·2 | 1·5 | — |
| M. S— | 16 | 12·3 | 10·5 (loculated) | <0·1 | 0·06 ,, ,, 8 | 3·7 | 1–1·5 | — |
| *Other consignments of $Cr^{32}PO_4$—* | | | | | | | | |
| | 18 | — | — | — | — | — | — | — |
| | 23 | — | — | — | — | — | — | — |
| | 20 | — | — | — | — | — | — | — |
| | 14 | — | — | — | — | — | — | — |

* Whole dose dialysed prior to administration.

*Specific concentration of $^{32}P$ in serous fluid*

The specific concentrations of $^{32}P$ in the serous fluid at 2 hours, 3–5 days and at longer intervals after administration of the $Cr^{32}PO_4$ are given in Table II in terms of $\mu$c./ml./10 mc. dose. In most cases the specific radioactivity fell to less than 10 per cent by the fourth day, a change which is much too great to be explained by any increase in volume of the serous fluid.

In four patients (M. F—, O. W—, L. S—, J. S—) the fall in specific radioactivity was much less marked and the reason for this variation is not understood.

The specific radioactivity of the serous fluid was generally much lower for peritoneal effusions than for pleural effusions.

*$^{32}P$ in blood*

The amount of $^{32}P$ circulating in the blood remained reasonably constant over the usual period of study of one week and showed only a slight fall in later samples taken as long as 26 days after administration of the $Cr^{32}PO_4$. The results are given in Table II. In patient J. B—, where dialysis of the $Cr^{32}PO_4$ dose was carried out prior to administration the concentration of $^{32}P$ in the blood was appreciably reduced.

The proportion of the circulating $^{32}P$ incorporated in the red cells builds up to 70–85 per cent over the first few hours after intracavity administration of $Cr^{32}PO_4$, after which it remained fairly constant for as long as measurements were made. The same pattern was followed after intravenous injection of $Na_2H^{32}PO_4$.

*Distribution of $Cr^{32}PO_4$. At autopsy*

The radioactive concentration ($Cr^{32}PO_4$) of various samples of pleura or peritoneum from the two cases which came to autopsy are given in Table III in terms of $\mu$c./sq.cm. per 10 mc. dose.

The striking feature of these results is the very wide variation of the order of 100 : 1. In case M. G—, it could be argued that the $Cr^{32}PO_4$ may have settled

TABLE III.—*Distribution of $Cr^{32}PO_4$ over the Serous Wall in Two Cases at Autopsy*

(Corrected to the time of administration)

Patient M. G—

Died 2½ months after $Cr^{32}PO_4$

Pleural effusion

| | $\mu$c./sq. cm. per 10 mc. dose |
|---|---|
| Pleura, upper lobe | 0·13 |
| Pleura (tumour) upper lobe | 0·12 |
| Parietal pleura (superior) | 0·17 |
| Parietal pleura (tumour) (inferior) | 0·43 |
| Parietal pleura (inferior) | 2·3 |
| Diaphragm | 17·0 |

Patient F. O'D—

Died 12 days after $Cr^{32}PO_4$

Peritoneal effusion

| | $\mu$c./sq. cm. per 10 mc. dose |
|---|---|
| Left post peritoneum | 0·38 |
| Right post peritoneum | 0·09 |
| Centre post peritoneum | 0·06 |
| Right lat. peritoneum | 1·1 |
| Left ant. peritoneum | 0·2 |
| Right ant. peritoneum | 0·5 |
| Left diaphragm peritoneum | 0·03 |
| Right diaphragm | 1·4 |
| Peritoneum over spleen | 0·1 |
| Wall of transverse colon | 3·6 |

under gravity to the lower regions of the pleural cavity, but there is no apparent pattern in the results of Case F. O'D—.

In Case M. G—, assay of the muscle underlying the pleura indicated a very marked fall off in radioactivity within a millimetre of the pleural surface.

*Results on experimental animals*

*Survival studies.*—All inoculated animals in both control and treated groups died. The mean survival for various groups is recorded in Table IV.

Single injections of $Cr^{32}PO_4$ in doses from 20 $\mu$c.–80 $\mu$c. per mouse failed to influence significantly the lethality from Ehrlich ascites tumour. When the dose was repeated (Group IX) and 75 $\mu$c. given on the third and seventh days of tumour

FIG. 5.—Survival curves for control inoculated mice injected with saline and inoculated mice receiving 2 doses of 75 $\mu$c. $Cr^{32}PO_4$ intraperitoneally, on 3rd and 7th day after inoculation, respectively (see text).

growth, the survival curve showed a biphasic trend, and nearly 50 per cent of animals died rapidly. apparently the result of the effects of total body irradiation (Fig. 5).

*Accumulation of Ascites Fluid.*—Colloidal $Cr^{32}PO_4$ in doses of 20–80 $\mu$c. given on the second day after inoculation caused a decrease in the usual gain in animal weight recorded over the first twelve days after inoculation with the tumour (Table IV, Groups II–V). A similar striking result was seen after the administration of two doses of 75 $\mu$c. on the third and seventh days of tumour growth (Group X).

This alteration in weight increase was due to a diminution in the accumulation of ascitic fluid. Measurements of daily fluid intake of treated and control mice revealed no essential difference in the daily consumption. For treated animal groups the mean daily fluid consumption per mouse for the first seven days after injection of $Cr^{32}PO_4$ ranged from 4·5 ml. to 6·9 ml., whilst control animals gave a corresponding range of 4·6 to 5·9 ml. This finding differs from that observed by van den Brenk and Parsons (1958) in the treatment of tumour inoculated mice with orally administered hydrogen peroxide, where the reduction in ascitic

TABLE IV.—*Effect of Colloidal $Cr^{32}PO_4$ Administered Intraperitoneally to Hybrid Walter and Eliza Hall Mice at Various Times after Inoculation with* $2 \times 10^6$ *cells of Ehrlich ascites Tumour*

| Group | Number of animals | Mean animal weight on day of inoculation (g.) | Mean survival time (± SD) | Mean survival time (Days) | Weight increase or loss over first 12 days after inoculation (g.) |
|---|---|---|---|---|---|
| I. Control (for Groups II–V) | 9 | 42·8 | 16·6 | (±2·1) | +8·5 |
| II. 20 μc. at 48 hours | 9 | 46·0 | 15·8 | (±1·9) | +1·1 |
| III. 40 μc. at 48 hours | 10 | 43·1 | 17·6 | (±3·1) | +0·1 |
| IV. 60 μc. at 48 hours | 10 | 46·5 | 17·4 | (±3·0) | −1·0 |
| V. 80 μc. at 48 hours | 10 | 41·5 | 19·0 | (±6·2) | −3·8 |
| VI. Controls (for Groups VII and VIII) | 10 | 28·1 | 18·6 | (±1·3) | — |
| VII. 20 μc. at 24 hours | 10 | 28·5 | 18·0 | (±1·5) | — |
| VIII. 20 μc. at 8 days | 10 | 29·0 | 16·6 | (±2·5) | — |
| IX. Control (for Group X) | 20 | 36·6 | 17·0 | (±3·6) | +13·1 |
| X. 2 × 75 μc. at 3 and 7 days | 19 | 37·1 | 15·3 | (±5·6) | +7·5 |

distension and body weight corresponded to reduced fluid intake and general dehydration of the treated animals.

Post-mortem examinations of $Cr^{32}PO_4$ treated mice showed a marked reduction of ascitic fluid, but large masses of tumour were adherent to mesenteries and abdominal viscera. The measurement of residual ascitic fluid *post-mortem* was an unsatisfactory determination owing to the interval which elapsed between death and the examination. Similarly the measurement of the ascitic fluid volume *in vivo* using dye dilution techniques was very unsatisfactory owing to turbidity of the fluids, the high contamination of the fluid with blood cells in many animals, and loculation of fluid in the treated mice in particular.

*Tumour cell population.*—Repeated measurements of the cell population in separate groups of control and treated animals showed a relative reduction in the number of tumour cells per unit volume ascitic fluid in the $Cr^{32}PO_4$ treated animals (Fig. 6). This reduction was particularly marked on the fourth day after administration of the $Cr^{32}PO_4$. Thereafter the tumour cell counts in all groups continued to fall, approaching a similar low level which was maintained until death of the animals.

Differential counts of non-viable cells, using the eosin technique, were made and in the control animals a sharp rise in the non-viable count occurred in the days immediately preceding death of the animals. The non-viable count was not raised in the isotope treated animals nor did it show the sharp rise before death recorded in control mice ; it would appear that the effect of the irradiation is to inhibit cellular proliferation.

### DISCUSSION

In most of the 16 cases studied the fall in the specific radioactivity of the serous fluid is far too great to be explained by an increase in its volume, a feature which has been observed by others (Root *et al.*, 1954), and since the body surveys indicate that most of the $^{32}P$ remains in the serous cavity it must be concluded that the $Cr^{32}PO_4$ plates out on to the serous wall. The rate at which this plating out process takes place varies considerably but in most cases it is nearly complete after a few days. The concentration of this $^{32}P$ over the serous wall however, has been shown from samples taken at two autopsies to vary very widely by a factor of 100 and determination of the dose of $Cr^{32}PO_4$ to be administered must therefore be empirical.

In this series no difference in dose has been made to allow for the smaller area of pleural cavities compared with peritoneal cavities, as suggested by Lange, Shields and Rozendaal (1956), or to allow for the various types of primary tumour. The size of the doses used for patients (Table I) shows no relation to the clinical results which followed. On the other hand, in the animal experiments where

FIG. 6.—Progressive tumour cell counts in ascitic fluid at various times after inoculation of mice with $2 \times 10^6$ cells Ehrlich ascites tumour, in control animals and animals treated with single intraperitoneal injections of $Cr^{32}PO_4$ 48 hours after inoculation.

conditions were better controlled a definite relationship was observed between dose and both fluid formation and cell counts.

Instability of a $Cr^{32}PO_4$ preparation not only reduces its efficacy as a source of local radiation of a serous cavity but may produce an unacceptable amount of $^{32}P$ in ionic form with rapid access to bone marrow.

On receipt of the $Cr^{32}PO_4$ about 15 per cent of the $^{32}P$ is found to be in ionic form and, as the material is dialysed prior to despatch from Amersham, this quantity must dissociate during the flight of approximately five days between England and Australia. The doses of $Cr^{32}PO_4$ used have been of the order of 10 mc. so the amount of ionic radioactive phosphate present amounts to about $1\frac{1}{2}$ mc. This is an undesirable feature and one that can be largely removed by dialysis of the material just prior to administration, but if facilities are not available for dialysis the amount of ionic radiophosphate present is not considered unacceptable for this type of case. To what extent further ionization of the $Cr^{32}PO_4$ continues after absorption of the colloid by the serous wall is not known, but the hazard of additional radioactive phosphate being absorbed is considered to be very small.

In animal experiments, not reported here, the effects of $^{198}Au$ and Nitramin on fluid formation and cell counts have been shown to be similar to those observed using $Cr^{32}PO_4$. This is consistent with reports of clinical experience with these agents (e.g. Hilton *et al.* 1957 ; Bonte, Storaasli and Weisberger, 1956) and the 8 out of 16 patients in the present series who showed marked clinical improvement following $Cr^{32}PO_4$ is a comparable result.

### CONCLUSION AND SUMMARY

It is considered that the use of radioactive colloidal chromic phosphate, as prepared by the Radiochemical Centre, Amersham, can be recommended for the treatment of malignant serous effusions since the hazard to the patient is small, particularly if the material is dialysed prior to administration. There are no toxic effects as experienced with cytotoxic drugs, and the hazard to staff handling the material and nursing the patient is negligible compared with radioactive colloidal gold.

The radioactive colloidal chromic phosphate was purchased by the National Welfare Fund of the Commonwealth Department of Health and procured by the Commonwealth X-ray and Radium Laboratory.

### REFERENCES

Bonte, F. J., Storaasli, J. P., and Weisberger, A. S.—(1956) *Radiology*, **67**, 63.
van den Brenk, H. A. S., and Parsons, I. C.—(1958) *Med. J. Aust.*, **1**, 633.
Dulbecco, R., and Vogt, M.—(1954) *J. exp. Med.*, **99**, 167.
Hilton, G., Halnan, K. E., Howard, N., and Godfrey, B. E.—(1957) *J. Fac. Radiol., Lond.*, **8**, 339.
Hoskins, J. M., Meynell, G. G., and Sanders, F. K.—(1956) *Exp. Cell. Res.*, **11**, 297.
Jaffe, H. L.—(1955) *Amer. J. Roentgenol.*, **74**, 657.
Lange, R. H., Shields, J. L., and Rozendaal, H. M.—(1956) *N.Y. St. J. Med.*, **56**, 1928.
Prentice, T. C., Siri, W., and Joiner, E. E.—(1952) *Amer. J. Med.*, **13**, 668.
Root, S. W., Tyor, M. P., Andrews, G. A., and Kniseley, R. M.—(1954) *Radiology*, **63**, 251.
Straube, R. L.—(1958) *Cancer Res.*, **18**, 57.